# Zinc Oxide Nanoparticles Induce DNA Damage in Sand Dollar *Scaphechinus mirabilis* Sperm

**DOI:** 10.3390/toxics10070348

**Published:** 2022-06-24

**Authors:** Sergey Petrovich Kukla, Victor Pavlovich Chelomin, Andrey Alexandrovich Mazur, Valentina Vladimirovna Slobodskova

**Affiliations:** Il’ichev Pacific Oceanological Institute, Far Eastern Branch, Russian Academy of Sciences, 690041 Vladivostok, Russia; chelomin@poi.dvo.ru (V.P.C.); mazur.aa@poi.dvo.ru (A.A.M.); slobodskova@list.ru (V.V.S.)

**Keywords:** ZnO NPs, genotoxicity, *Scaphechinus mirabilis*, comet assay

## Abstract

Products containing nanomaterials are becoming more and more common in everyday life. Zinc oxide nanoparticles (ZnO NPs), meanwhile, are among the most widely used NPs. However, their genotoxic effect on the germ products of marine organisms is poorly understood. Therefore, the effects of ZnO NPs and zinc ions (20, 50, 100, 200 µg/L) on the sperm of sand dollar *Scaphechinus mirabilis* were compared. Comet assay showed that both tested pollutants caused an increase in DNA damage to 6.57 ± 2.41 and 7.42 ± 0.88% DNA in the comet tail, for zinc ions and ZnO NPs, respectively. Additionally, a different pattern was shown by the increase in DNA damage, with increasing concentration of pollutants, in different experimental groups.

## 1. Introduction

A huge variety of nanomaterials have now been developed and are widely used in various industries (electronics, energy, construction, semiconductors, paint and varnish, household chemicals, etc.) and their number continues to grow rapidly [1,2,3]. The extent of nanomaterial penetration in consumer products widely used by humans in everyday life is particularly impressive: in medicine, clothing, detergents, perfumes, cosmetics, household items, as well as in food and personal care products [4,5,6].

The growing interest in nanoparticles (NPs) is due to the manifestation of new, unique physical and chemical properties in the transition of traditional materials to the nanodispersed state [7]. This makes scientists pay special attention to the ecotoxicological effects associated with NPs. It is these properties that make NPs highly reactive, forming bonds of a different nature with basic biostructures and cellular macromolecules, which may result in unique pathobiochemical effects. Current nanotoxicological research shows that NPs of a different nature are capable of crossing the body’s protective barriers: the gastric, placental and blood-brain barriers [8,9,10], penetrating biological membranes and accumulating in biological systems of different levels of organization [8,11,12,13]. This shows that complex ecotoxicological problems associated with the introduction of nanotechnology products into the biosphere and the danger of different composition NPs interfering with biochemical processes in the living organism are inevitable.

Products of nanotechnology inevitably enter the environment in different ways: through production, processing, transport, use and disposal. In doing so, they inevitably end up in coastal ecosystems due to various migration processes [14,15]. Therefore, not only humans, but also the environment with all of its biodiversity, are becoming increasingly influenced by NPs. 

The penetration of NPs into the marine environment is fraught with numerous consequences which, due to lack of information, cannot yet be predicted. Despite the low water solubility and tendency for aggregation of NPs, particularly metal oxides [16], which to some extent limits their accessibility to marine organisms, experimental data indicate high levels of NPs accumulation in various species of marine organisms, such as crustaceans, echinoderms and mollusks [14,17,18].

Among a wide range of metal oxide NPs, ZnO NPs are considered to be among the most common used [19]. Due to their unique characteristics, ZnO NPs are widely used in instrumentation (including bioelectronics and biomedical devices), cosmetics, pharmaceuticals, UV filters, biomaterials and food packaging materials.

In the literature, there are sufficiently convincing data demonstrating the effect of ZnO NPs on various functional aspects of marine organisms [20,21,22]. A number of studies have revealed a higher toxicity of ZnO NPs, not only in relation to those that are identical in size to particles of oxides of other metals [23,24], but also in comparison with zinc ions [20,25]. Of increased concern to ecotoxicologists is the ability of ZnO NPs to induce genome damage, manifested in impaired gene expression in mussels [26], and in chromosomal aberrations and morphological changes in the gametes and embryos of sea urchins *Paracentrotus lividus* [20,27]. In addition, in the example of the sea urchin *Scaphechinus mirabilis*, the exposure of adults to ZnO NPs has been shown to affect the reproductive system, causing DNA damage in gametes and abnormalities in larval development [25,28].

The increased ecotoxicological interest in the early stages of development is due to the generally accepted view in the literature that gametes, embryos and larvae are more sensitive than adults and that they represent a critical period in the life cycle of an organism [29,30]. Spermatozoa are highly specialized cells and, unlike somatic cells, they are potentially more susceptible to damage by substances exhibiting genotoxic properties, as they contain highly condensed DNA, have weak antioxidant protection and very limited ability to repair DNA damage [31,32,33,34]. Therefore, spermatozoa are very vulnerable to oxidative stress, which is known to be one of the main mechanisms involved in DNA damage.

*S. mirabilis*, as a typical member of the sea urchin class, releases sexual gametes directly into the sea water during spawning, where fertilization and further development of embryos and larvae occurs. At this stage, spermatozoa are the least protected and their outer membranes and receptors are directly exposed to a wide range of chemicals [35,36]. In this respect, our experiments mimic environmental conditions in terms of Zn^2+^ and ZnO NPs interaction with sea dollar gametes. Our studies were carried out under controlled laboratory conditions with no exposure to other concomitant stressors typical of the marine environment. Zn^2+^ and ZnO NPs concentrations, which are widely used in ecotoxicological experiments, were used [37]. This approach, which belongs to the category of “acute” experiments, aims to identify likely “targets”, i.e., most vulnerable cell structures, and associated toxicity mechanisms. In order to assess the potential danger posed by the penetration of nanoparticles into the marine environment, we investigated the genotoxic properties of NPs in male sea urchin gametes. 

To determine genotoxicity, we applied the comet assay method, a sensitive method for the early detection of DNA damage [38], which is widely used in research on the toxicity of NPs [39]. 

The relevance of assessing DNA damage in sperm cells increases dramatically, given that DNA damage to these cells typically has deleterious effects on species reproduction (reproductive success), which is an important indicator of long-term ecotoxicological consequences [31].

Based on the above, the aim of this work was to investigate, using the spermatozoa of the sea urchin *S. mirabilis*, the potential risk posed by ZnO NPs to the genome integrity of marine invertebrate gametes.

## 2. Materials and Methods

### 2.1. Preparation of Working Solutions

Stock solution containing zinc ions was prepared using zinc chloride. ZnO NPs solution was prepared using commercial NPs (Sigma-Aldrich (Darmstadt, Germany), particle size <50 nm; Cat #677450). The main properties of the NPs were discussed in Tang et al., (2018) [40] and are presented in Table 1.

### 2.2. Description of the Experiment

Adult sea dollars were collected in Peter the Great Gulf, Japan Sea and delivered to the laboratory within one hour. After delivery, the urchins were acclimatized for 2 days in water filtered by a three-stage gravel filter and sterilized by ultraviolet (pH 8.2; salinity 32.75 ppm, O_2_ concentration 7.5 ± 0.3 mg/L, T = 17–18 °C).

Semen from 4 males *S. mirabilis* in 2 replicates was used in an experiment. Spermatozoa were obtained by stimulating spawning with 0.5 M potassium chloride solution. Semen was collected immediately before the experiment and diluted with pure seawater. Both experimental and control groups used semen from the same males.

To study genotoxicity, experimental solutions were added to the diluted semen aliquot to final concentrations of 20, 50, 100 and 200 µg Zn/L and incubated for 1 h [41]. After that, it was used in the comet assay.

### 2.3. Comet Assay

An alkaline version of the comet assay adapted for marine organisms was used [42]. 

First, 50 µL of semen suspension was added to 100 µL of 1% fusible agarose in 0.04 M phosphate buffer (pH 7.4) at 37 °C, thoroughly mixed, and applied on a slide coated with 1% agarose solution for better adhesion and covered with a coverslip. The sample was placed in the refrigerator for 3 min to form a gel. The coverslip was carefully removed and the slide was submerged into the lysis solution (2.5M NaCl; 0.1M EDTA-Na2, 1% Triton X-100; 10% DMSO; 0.02 M Tris, pH 10) for 1 h in the dark at 4 °C. After washing with distilled water, the slides were placed in electrophoresis buffer (300 mM NaOH, 1 mM EDTA-Na2) and incubated for 40 min. Electrophoresis was performed at 2 V/cm for 15 min. After neutralization (0.4 M Tris-HCl, pH 7.4), the slides were stained with SYBR Green fluorescent dye.

DNA comets were visualized and recorded using a fluorescence microscope (Zeiss, Axio Imager A1) equipped with an AxioCam MRc digital camera. For digital image processing, the CaspLab computer program was used to calculate various comet parameters indicating the degree of cellular DNA damage. At least 50 comets were analyzed for each glass. For each comet, the proportion of DNA in the comet tail (% of DNA in tail) was determined.

### 2.4. Statistical Analysis

Statistical processing of the results was performed using STATISTICA 8 software (StatSoft, Tulsa, OK, USA). The significance of the differences between the control and experimental groups was assessed by means of a one-factor analysis of variance using Dunnett’s test (at *p* ≤ 0.05).

## 3. Results and Discussion

Evaluation of DNA damage after exposure of experimental solutions gave the following results.

**Figure 1:**
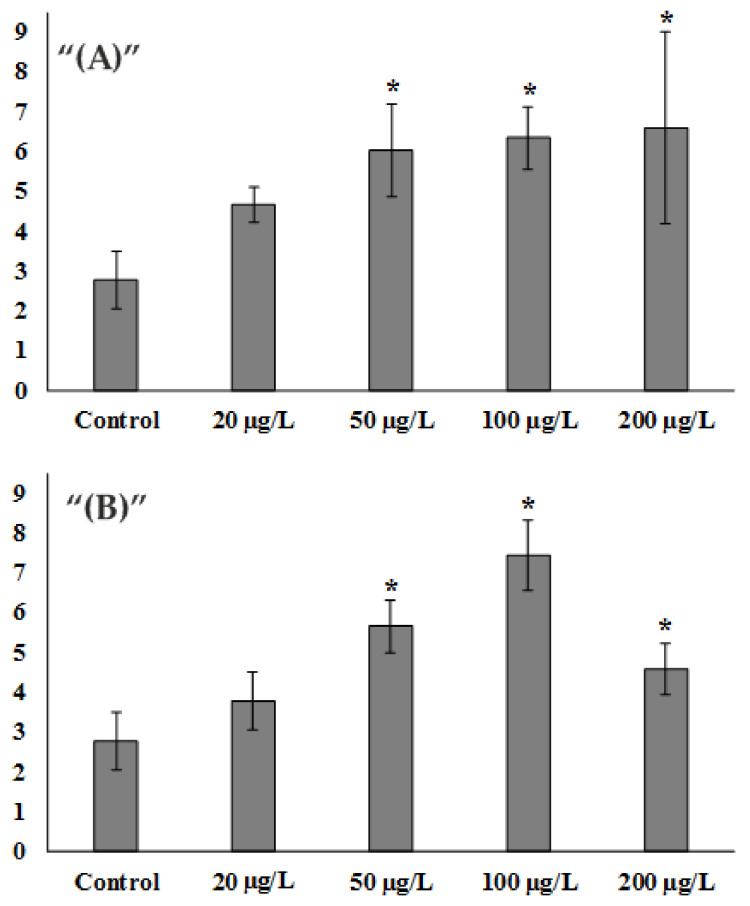
Assessment of *S.mirabilis* sperm DNA damage from control and experimental groups. (**A**) Zn^2+^ exposure. (**B**) ZnO NPs exposure. (Mean ± standard deviation, N = 8, n = 400.) * Difference from the control is significant (*p* < 0.05).

After experimental exposure to Zn^2+^, already at the concentration of 20 μg/L, there was an increase in DNA damage by about 1.6 times which amounted to 4.68 ± 0.44% DNA in the tail, compared to the control group, where the damage percentage was 2.77 ± 0.73% DNA in the tail. At concentrations of 50 µg/L and higher, a statistically significant 2.1-fold increase in DNA damage was observed, corresponding to 6.04 ± 1.17% DNA in the tail. At the same time, a further increase in zinc ions in water up to the values of 100 µg/L and 200 µg/L did not lead to a further sharp increase in DNA damage and exceeded the control value by 2.2- and 2.3-fold, respectively (6.33 ± 0.78 6.57 ± 2.41% DNA in the tail) (Figure 1A). 

When exposed to low concentrations of ZnO NPs, a similar pattern to that of zinc ions was observed. Exposure to 20 µg/L resulted in a slight increase in DNA damage of approximately 1.3-fold and amounted to 3.77 ± 0.74% DNA in the tail. At 50 µg/L, the difference was more than 2-fold and amounted to 5.65 ± 0.66% DNA in the tail. At 100 µg/L there was a further 2.6-fold increase in DNA damage to values of 7.42 ± 0.88% DNA in the tail. Nevertheless, at the NPs concentration of 200 μg/L, there was a decrease in the extent of DNA damage. It was only 1.6-fold higher than the control and amounted to 4.59 ± 0.66% DNA in the tail (Figure 1B).

In order to estimate the size of the detected destructive changes in sperm exposed to ZnO NPs, one can refer to the results of Lacaze et al. (2011) [32]. Using healthy amphipods of *Gammarus fossarum* as an example, they determined the reference and threshold values of sperm genome damage with regard to seasonal variations. The reference value of DNA damage in sperm was found to be 3.1%, while the minimum and maximum threshold values were 2.6% and 3.5%, respectively [32].

Comparison of these data with our results obtained on sand dollar sperm suggests that exposure to ZnO NPs leads to severe damage in the sperm DNA molecule at all concentrations studied.

Literature data on the genotoxic properties of NPs are still scarce. Similar studies using sea urchin sperm of *Paracentrotus lividus* have also revealed genotoxic properties of ZnO and CuO NPs [27,43]. In addition, the genome sensitivity of male gametes to various nanoparticles has been shown in other marine invertebrates, including the bivalve *Tegillarca granosa* [44], the polychaete *Hydroides elegans* [45] and the ascidia *Ciona intestinalis* [46].

In explaining the mechanisms of toxicity, the view has developed and become popular that the toxicity of NPs is directly related to Zn^2+^, which is formed due to the instability of NPs in the aqueous medium [47]. However, given the short-term nature of experiments in this research, it is logical to assume that most of the ZnO NPs retain their structure and the Zn^2+^ concentration is very low [47]. Thus, the main contribution to DNA degradation is made by NPs themselves. In addition, several previous studies have revealed differences in ecotoxicological effects initiated by NPs and zinc ions [20,28]. Oliviero et al. (2019) drew attention to the lack of a direct correlation between levels of sperm DNA damage and NPs concentration [27]. They noted that, in a certain range of changes in ZnO NP concentrations, a gradual increase in the number of damaged “comets” was observed, reaching a maximum value (3 μM, in their case), but with a further increase in concentration, this rate began to decrease gradually, in contrast to exposure to dissolved zinc. Additionally, a similar dependence was observed in the analysis of the number of skeletal anomalies in sea urchin larvae of *P. lividus* after exposure to different concentrations of TiO_2_ NPs [48]. This pattern in our results and those reported in the literature can be explained by the fact that the aggregation rate of NPs depends on their concentration in the aqueous medium [49]. At higher concentrations, nanoparticles, particularly ZnO NPs, tend to form large aggregates, which become less bioavailable and, consequently, cause less effect.

The mechanisms underlying the genotoxic effects of ZnO nanoparticles on male gametes are not clear. Most researchers, in explaining the causes of genotoxicity, draw attention to the ability of ZnO to induce increased generation of reactive oxygen species (ROS), thereby causing oxidative stress. Highly reactive oxyradicals are thought to be the main cause of oxidative damage and DNA strand breaks [11]. Additionally, the possibility of NPs directly affecting gene structures and their regulation mechanisms cannot be ruled out [50].

It has been established that the main modes of entry of NPs into a living cell are variants of endocytosis, in particular through receptor-mediated pinocytosis [51]. At present, there are no data on the penetration of NPs into sperm cells. Given the adsorption characteristics of NPs and their ability to interact with cellular receptors responsible for the transmission of stress signals [6], the following suggestion can be made. In the framework of oxidative stress, it can be suggested that ZnO NPs, when located at different sites of the outer sperm membrane, can disorganize the receptor-signaling system to some extent and induce the formation of ROS. As shown earlier, sea urchin spermatozoa are capable of generating several types of ROS, such as H_2_O_2_ and O_2^−^_ [52]. It is likely that a similar mechanism of ROS formation was initiated through the exposure of *P. lividus* sea urchin sperm to CuO NPs, causing significant DNA fragmentation [46]. Additionally, was showed that TiO_2_ NPs, when interacting with gills, caused oxidative stress without penetrating cells [53].

In the context of the problem at hand, it must be taken into account that spermatozoa and oocytes have a unique and essential biological function in forming the genome for the development of the next generation. Therefore, the integrity of gamete genomes is of paramount importance for the development of viable offspring.

However, the biochemical response of the organism, such as DNA damage, most often outpaces the cytological response. For example, short-term exposure of bivalve spermatozoa to benz[a]pyrene and diuron resulted in significant and dose-dependent DNA damage, but the sperm retained fertilizing capacity [31,54]. Additionally, in other studies, despite the high level of DNA damage in fish sperm induced by exposure to genotoxicants, fertilization success was maintained at a high level [34,55,56].

It is thought that, after fertilization, damaged paternal DNA may be partially repaired by the embryo repair system [34,57], or serve as a signal to trigger various developmental anomalies and embryonic deaths.

Indirect support for this assumption is provided by numerous laboratory studies in recent years that have demonstrated a relationship between sperm DNA integrity and offspring quality in aquatic organisms [31,32,55,56,57]. For example, rainbow trout eggs fertilized with sperm with varying degrees of fragmented DNA formed embryos with lower chances of survival [57]. Numerous malformations, mainly in skeletal development, were observed in the hatching larvae of the three-legged stickleback, grown from eggs fertilized with methylmethanesulfonate-treated spermatozoa [56].

In conclusion, we believe that despite the relatively low degree of DNA molecule destruction in spermatozoa detected in our experiments, the risk of further initiation of destructive processes and manifestation of long-term undesirable effects remains. Further research in this area should focus on a detailed study of the biochemical mechanisms involved in sperm DNA damage.

## Figures and Tables

**Table 1:** The basic properties of ZnO NPs.

Purity, %	Zeta Potential, mV	Particle Size, nm	Hydraulic Radius, nm	BET, m^2^/g
≥99.5	−39.4	40–50	200	58

## Data Availability

Not applicable.
